# Yeast cell wall polysaccharides accelerate yet in-feed antibiotic delays intestinal development and maturation via modulating gut microbiome in chickens

**DOI:** 10.1186/s40104-024-01145-x

**Published:** 2025-01-25

**Authors:** Fangshen Guo, Jianing Qiao, Zeqiong Hu, Jia Huang, Ruichen Bi, Waseem Abbas, Wenrui Zhen, Yuming Guo, Zhong Wang

**Affiliations:** 1 State Key Laboratory of Animal Nutrition, College of Animal Science and Technology, China Agricultural University, Beijing, 100193 People’s Republic of China; 2 Henan International Joint Laboratory of Animal Welfare and Health Breeding, College of Animal Science and Technology, Henan University of Science and Technology, Luoyang, 471000 People’s Republic of China

**Keywords:** Antibiotic, Broiler chickens, Gut development, Microflora, Yeast cell wall polysaccharides

## Abstract

**Background:**

It is important to promote intestinal development and maturation of chicks for feed digestion and utilization, intestinal health, and disease resistance. This study aimed to investigate the effects of dietary yeast cell wall polysaccharides (YCWP) addition on intestinal development and maturation of chickens and its potential action mechanism.

**Methods:**

180 one-day-old male Arbor Acres broilers were randomly assigned to three groups containing control (basal diets without any antibiotics or anticoccidial drug), bacitracin methylene disalicylate (BMD)-treated group (50 mg/kg) and YCWP-supplemented group (100 mg/kg).

**Results:**

Compared with control group, in-feed antibiotic BMD continuous administration significantly decreased crypt depth (d 21) and villus height (d 42) along with mucosal maltase activity (d 42) in the ileum (*P* < 0.05). Also, BMD markedly downregulated gene expression levels of β-catenin, lysozyme, occludin and *FABP-2* (d 21) and innate immune related genes *CD83* and *MHC*-*I* mRNA levels (d 42, *P* < 0.05), and decreased goblet cell counts in the ileum of chickens (d 21 and d 42,* P* < 0.05). While, *TLR-2*, *TLR-6* and *iNOS* mRNA abundances were notably upregulated by BMD treatment (d 42, *P* < 0.05). Nevertheless, dietary YCWP addition significantly increased the ratio of villus height to crypt depth (d 21), villus surface area (d 21 and d 42), ileal alkaline phosphatase and maltase activities as well as goblet cell (d 21 and d 42) and IgA-producing plasma cell numbers as compared to BMD treatment (d 21, *P* < 0.05). YCWP addition also upregulated gene expression levels of *Lgr5*, Wnt/β-catenin signaling pathway related gene (Wnt3, β-catenin, d 21; β-catenin, d 42), intestinal cells proliferation marker *Ki-67* and barrier function related genes (occludin, d 21 and d 42, *P* < 0.05). Moreover, YCWP significantly increased antigen presenting cell marker related genes (*MHC*-*II*, d 21; *CD83* and *MHC*-*I*, d 42), *TLR-1*, *TLR-2* and *TLR-6* mRNA levels (d 21, *P* < 0.05). Cecal microbiome analysis showed that YCWP addition obviously improved cecal microbial composition, as indicated by increasing relative abundance of *Fournierella*, *Psychrobacter* and *Ruminiclostridium* on d 21, and *Alistipes* and *Lactobacillus* on d 42, which were positively related with gut development and maturation related indexes (*P* < 0.05).

**Conclusion:**

Collectively, YCWP promoted yet antibiotic BMD delayed intestinal morphological and immunological development linked with modulating gut microbiome in chickens.

**Supplementary Information:**

The online version contains supplementary material available at 10.1186/s40104-024-01145-x.

## Introduction

Gut is a highly complex system composed of intestinal epithelium layer, intestinal microbes, and mucosal immune system, which are intertwined with each other to influence the growth, development, maturity, health along with other biological function of gut [1]. The morphological structure, microbial composition along with mucosal immune system of the early chicken gastrointestinal system develop slowly [2]. This immature intestinal immune function could increase the susceptibility to pathogenic microorganisms in the growing stage of chickens, which might reduce growth performance and eventually lead to heavy financial losses in intensive production [3]. To control enteric pathogenic bacteria infections, antibiotics are commonly used in the chicken industry. However, impeded intestinal stem cell (ISC) activity and intestinal development [4], insulted gut barrier function [5], compromised intestinal mucosal immunity and vaccine efficacy as well as increased disease susceptibility [6], disrupted intestinal microbial community composition [7], had been reported in animals with prolonged antibiotics exposure. Foremost, accumulating evidences had shown that the use of antibiotics in poultry and livestock industry is associated with many adverse effects such as the emergence of antibiotic-resistant bacteria and antibiotic residues in animal product, which bring serious public concerns for human health [7, 8]. Therefore, it is of great significance to develop non-antibiotic strategies to promote the development and maturation of intestinal morphology, microbiota and immune functions of early chickens.

Development and self-renewal of intestinal epithelial cells were derived from original leucine-rich repeat-containing G protein-coupled receptor 5 positive (Lgr5^+^) ISCs harbored at the bottom of crypt of the gut. Lgr5^+^ ISCs can give rise to daughter or progenitor cells, which can subsequently differentiate into the mature cell types such as intestinal columnar epithelial cells, goblet cells, endocrine cells and Paneth cells, required for normal gut function [9, 10]. Wnt/β-catenin and Notch pathways synergistically control the direction of intestinal stem cell proliferation and differentiation into intestinal epithelial cells [11]. Additionally, gut microbiota of chickens also plays a critical role in gut morphological and immunological development, digestion and absorption, disease resistance [12–14]. To date, studies had reported that certain factors such as nutrients, probiotics and prebiotics, had obvious impacts on the intestinal development and maturity via modulating Wnt/β-catenin signaling pathway, intestinal stem cell proliferation and differentiation activity together with intestinal microbial composition in chickens [15].

Yeast cell wall polysaccharides (YCWP) extracted from the cell wall of *Saccharomyces cerevisiae* are mixed polysaccharides, which mainly contain β-glucan and mannan, and a small amount of chitin. Previous studies had demonstrated that yeast-derived mannan oligosaccharides and β-glucans can be recognized by pattern recognition receptors expressed on macrophages and dendritic cells, which leaded to the activation of innate immune system, resulting in increased macrophages phagocytosis activity, production of cytokines and antimicrobial peptides, as well as enhanced innate and acquired immunity function [16, 17]. In poultry industry, YCWP has been widely used as antibiotics alternative, growth promotors and immuno-modulators [18], for its beneficial biological activities, such as growth-promoting, immune-regulation [19–22], barrier-improvement [23, 24], anti-inflammation [25, 26], anti-infection [23, 27–32] and mycotoxin-absorption [33, 34]. However, the regulatory effects and underlying mechanism of YCWP on intestinal development and maturity of chickens has not been investigated. The objective of current study was to first investigate the effects of YCWP on gut development and maturity by determining intestinal morphology, intestinal mature functional cells biomarkers, intestinal innate immune responses and barrier functions related gene expression through comparing with in-feed antibiotic bacitracin methylene disalicylate (BMD) administration, and then the underlying action mechanism of YCWP regulating gut development of chickens was further explored via measuring intestinal microbial composition and Wnt/β-catenin signaling pathway related gene abundance.

## Materials and methods

### Experimental design and diets

A total of 180 one-day-old healthy male Arbor Acres broilers with similar body weight were randomly assigned into 3 treatment groups. Each group contained 6 replicates with 10 birds per replicate. Birds were fed a corn-soybean-based diet (C), antibiotic-supplemented diet with BMD at 50 mg/kg diet (A), and YCWP-added diet supplemented with 100 mg YCWP per kg diet (Y), respectively. The pelleted basal diet was formulated in accordance with the recommendations of the American National Research Council (NRC, 1994) [35]. The composition and nutrient levels of basal diet are presented in Table 1. All birds were allowed ad libitum access to feed and water. The experiment lasted for 42 d. The YCWP used mainly consists of β-glucan (18.0%) and mannan (21.5%) and other components, which was provided by Arm Hammer Animal and Food Production, Church & Dwight Co., Inc. (USA).

**Table 1:** Composition and nutrient levels of basal diet, % (as-fed basis)

Ingredients	d 1–21	d 22–42
Corn (CP 8.0%)	53.02	53.27
Soybean meal (CP 46.0%)	34.00	33.50
Soybean oil	4.00	5.00
Wheat powder (CP 13.8%)	5.00	5.00
Limestone	1.18	1.00
Calcium hydrogen phosphate	1.70	1.20
DL-Methionine (98%)	0.20	0.15
L-Lysine sulfate (78%)	0.20	0.20
Choline chloride (50%)	0.20	0.15
Phytase	0.02	0.01
Sodium chloride	0.25	0.35
Mineral premix^a^	0.15	0.10
Vitamin premix^b^	0.03	0.02
Antioxidants^c^	0.05	0.05
Total	100.00	100.00
Nutrient levels^d^		
Metabolizable energy, Mcal/kg	3.02	3.10
Crude protein, %	19.94	19.48
Ethanol extract, %	5.61	3.02
Dry matter, %	91.25	87.22
Total calcium, %	0.95	0.76
Total phosphorus, %	0.68	0.59
Lysine, %	1.22	1.21
Methionine, %	0.50	0.44

^a^Mineral premix provided per kilogram of complete diet: iron, 80 mg; copper, 8 mg; manganese, 100 mg; zinc, 80 mg; iodine, 0.35 mg; selenium, 0.15 mg

^b^Vitamin premix provided per kilogram of complete diet: vitamin A (retinyl acetate), 12,500 IU; vitamin D_3_ (cholecalciferol), 2,500 IU; vitamin E (DL-a-tocopherol acetate), 30 IU; vitamin K_3_ (menadione sodium bisulfate), 2.65 mg; vitamin B_12_ (cyanocobalamin), 0.025 mg; biotin, 0.30 mg; folic acid, 1.25 mg; nicotinic acid, 50 mg; D-pantothenic acid, 12 mg; pyridoxine hydrochloride, 6.0 mg; riboflavin, 6.5 mg; thiamine mononitrate, 3.0 mg

^c^Antioxidant: 33% ethoxyquinoline

^d^Crude protein, ethanol extract and dry matter are measured values and others are calculated values based on the analyzed data of experimental diets

### Sample collection

On 21 d of age (d 21) and d 42, one bird from each replicate were randomly selected, weighed, and euthanized by cervical dislocation. The middle segments of ileum were cut off, rinsed with ice-cold sterile saline and fixed in 4% paraformaldehyde for morphology and immuno-histochemistry analysis. The ileal mucosa was collected, nap-frozen in liquid nitrogen, and then stored at −80 °C for the further analysis of gene expression and enzyme activity. The cecal content samples were collected and froze immediately in liquid nitrogen and then transferred to −80 °C for 16S rDNA analysis.

### Enzyme activity measurements

Ileal mucosa samples were weighted, diluted in sterile cold physiological saline (w/v, 1:9), homogenized and then centrifuged (3,000 × *g*, 4 °C, 10 min) to collect the supernatant. The activities of intestinal maltase, sucrase, and alkaline phosphatase (ALP) and the protein content in mucosal supernatant were measured using commercial kits from Nanjing Jiancheng Bioengineering Institute (Nanjing, China).

### Intestinal morphology

Villus height, crypt depth and villus width of the ileum were analyzed as previously described [36]. The ratio of villus height to crypt depth and the villus surface area (VSA = π × villus height × villus width) were calculated.

### Alcian Blue and periodic acid-Schiff staining

The density of goblet cells in the intestine were measured as previously described [36]. Goblet cells producing acidic and neutral mucins were identified by Alcian Blue (AB) and periodic acid-Schiff (PAS) staining.

### Immunohistochemistry assay

The number of immunoglobulin A (IgA)-positive cells in the ileum was measured using immunohistochemistry method according to the previous protocol [36].

### Quantitative real time PCR

Total RNA of ileal samples was extracted using RNAiso Plus (Takara, Dalian, China) and then treated with the PrimeScript RT reagent Kit with gDNA Eraser (Takara, Dalian, China) to remove the genomic DNA. RNA concentration and purity were determined using a NanoDrop 2000 spectrophotometer (Thermo Fisher Scientific, Waltham, MA, USA). Complementary DNA was synthesized using 2 μg of total RNA using PrimeScript™ RT reagent Kit (Takara, Dalian, China) according to the manufacturer's instructions. Quantitative real-time PCR (RT-qPCR) reactions were performed in the Applied Biosystems 7500 Fast Real-Time PCR system (Thermo Fisher Scientific, MA, USA) by using SYBR Premix Ex Taq diagnostic kit (Takara, Dalian, China) and each sample was measured in duplicate. The cycling conditions were as follows: 95 °C for 20 s, then 40 cycles of 90 °C for 3 s and 60 °C for 30 s. All data were analyzed using the 2^−∆∆CT^ method, and normalized by β-actin gene expression. Primer sequences are listed in Table S1.

### Microbial genomic DNA extraction and 16S rRNA gene sequencing

Microbial genomic DNA was extracted from cecal contents using QIAamp Fast Stool Mini Kit (Qiagen, Hilden, Germany) according to the manufacturer’s instructions. NanoDrop 2000 (Thermo Fisher Scientific, Waltham, MA, USA) was used to determine the concentration of DNA, and 1% agarose gel electrophoresis was used to assess the purity of DNA. The common primers 338 F (5’-ACTCCTACGGGAGGCAGCA-3’) and 806 R (5’-GGACTACHVGGGTWTCTAAT-3’) targeting the V3–V4 region of the 16S rDNA gene were used to amplify bacterial DNA. PCR products were purified, quantified and homogenized to construct a sequencing library. Amplicon libraries were sequenced on the Illumina MiSeq PE250 platform (Illumina, CA, USA) at Shanghai Personal Biotechnology Co., Ltd. (Shanghai, China). The sequences were merged by FLASH, and quality-filtered by Trimmomatic. Subsequently, UCHIME was adopted to remove the chimeric sequences, obtaining the effective tags, and further analyzed by the QIIME software (version 1.8.0). The effective reads were clustered into operational taxonomic units (OTUs) based on the 97% similarity. Afterward, basing on the Silva taxonomic database, OTUs were annotated. Alpha-diversity indexes were investigated by Mothur (version 1.30), and the significance was determined using a Mann–Whitney U test. β-Diversity was conducted using binary_jaccard distance (PERMANOVA/ANOSIM analysis) in QIIME software. The linear discriminant analysis (LDA) effect size (LEfSe) was adopted to identify the differential taxa abundance between groups based on the taxonomic files obtained from the QIIME analysis.

### Statistical analysis

Data were subjected to ANOVA after determination of variance homogeneity by using the SPSS 17.0. Data were expressed as mean and pooled SEM. Significant differences among groups were determined by one-way ANOVA followed by Duncan’s multiple-range test. A value of *P* ≤ 0.05 was considered significant, while value of *P* at 0.05 to 0.10 was classified as trends. Correlations were analyzed using spearman correlation with the p-heatmap package.

## Results

### Body weight and gut morphology

**Fig. 1:**
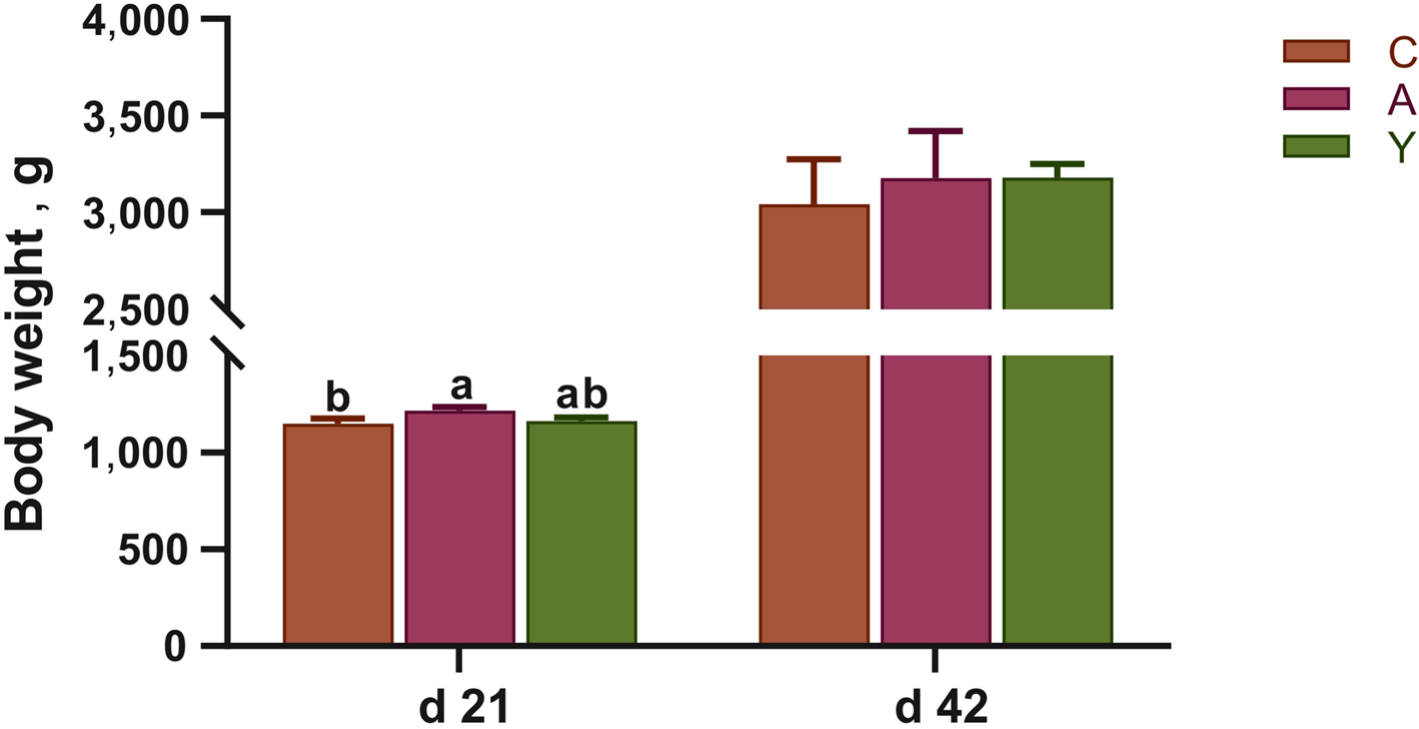
Effect of yeast cell wall polysaccharides on body weight of chickens. ^a,b^Different letters on the bar charts indicate significant differences (*P* < 0.05, *n* = 6). C: Broiler chickens fed with basal diet. A: Broiler chickens fed with the basal diet supplemented with 50 mg/kg bacitracin methylene disalicylate. Y: Broiler chickens fed with basal diet supplemented with 100 mg/kg yeast cell wall polysaccharides

As shown in Fig. 1, antibiotic BMD increased the body weight of chickens at d 21 (*P* < 0.05). As exhibited in Table 2, compared with control group, BMD obviously shortened crypt depth while increased villus width and the ratio of villus height to crypt depth on d 21 (*P* < 0.05). On d 42, BMD significantly reduced villus height on d 42 as compared to the control (*P* < 0.05). However, relative to BMD group, YCWP notably increased villus height at d 21 and villus surface area at d 42 (*P* < 0.05) but showed a lifted trend for crypt depth at d 21 and villus surface area at d 21 and d 42, which was very similar to the changes of the control group. Furthermore, YCWP administration had higher ratio of villus height to crypt depth and villus surface area than the control chickens at d 21 (*P* < 0.05).

**Table 2:** Effect of yeast cell wall polysaccharides on the ileal morphology of broilers

Items	C	A	Y	SEM	*P*-value
d 21					
Villus height, μm	1,456.75^ab^	1,318.49^b^	1,564.77^a^	46.390	0.048
Crypt depth, μm	303.76^a^	233.41^b^	256.78^ab^	11.130	0.020
Ratio of villus height to crypt depth	4.84^b^	5.69^a^	6.11^a^	0.194	0.015
Villus width, μm	382.47^b^	466.23^a^	408.08^ab^	15.932	0.017
Villus surface area, mm^2^	1.62^b^	1.91^ab^	2.00^a^	0.069	0.049
d 42					
Villus height, μm	1,733.96^a^	1,364.84^b^	1,576.49^ab^	63.679	0.049
Crypt depth, μm	257.65	235.23	251.81	7.103	0.490
Ratio of villus height to crypt depth	6.74	5.87	6.30	0.218	0.428
Villus width, μm	428.51	452.29	490.56	18.932	0.280
Villus surface area, mm^2^	2.20^ab^	1.93^b^	2.70^a^	0.126	0.030

C: Broiler chickens fed with basal diet. A: Broiler chickens fed with the basal diet supplemented with 50 mg/kg bacitracin methylene disalicylate. Y: Broiler chickens fed with basal diet supplemented with 100 mg/kg yeast cell wall polysaccharides

^a,b^In the same row, values without common superscripts differ significantly (*P* < 0.05, *n* = 6)

### Ileal disaccharidase and ALP activities

Intestine-specific ALP and brush border disaccharidase are positively involved in gut development, maturation and homeostasis. As exhibited in Table 3, BMD notably decreased the activity of maltase of chicken at d 42 (*P* < 0.05), compared with the control group, indicating a delayed gut maturity induced by antibiotic BMD. However, YCWP remarkably increased ALP activity comparing with control group and the BMD group at d 21, and exhibited greater maltase activity relative to the BMD treatment at d 21 and d 42 (*P* < 0.05), indicating a notable facilitated effect of YCWP on gut development and maturity of chickens.

**Table 3:** Effects of yeast cell wall polysaccharides on ileal disaccharidase and alkaline phosphatase activity of broilers

Items	C	A	Y	SEM	*P*-value
d 21					
Sucrase, U/mg protein	13.77	11.69	20.24	2.180	0.260
Maltase, U/mg protein	95.35^ab^	62.31^b^	115.94^a^	9.140	0.043
ALP, U/g protein	1.40^b^	1.69^b^	7.15^a^	0.704	< 0.001
d 42					
Sucrase, U/mg protein	40.33	30.98	43.39	2.762	0.163
Maltase, U/mg protein	117.43^a^	81.07^b^	112.43^a^	5.787	0.011
ALP, U/g protein	7.62	10.20	11.53	0.815	0.136

*ALP* Alkaline phosphatase. C: broiler chickens fed with basal diet. A: Broiler chickens fed with the basal diet supplemented with 50 mg/kg bacitracin methylene disalicylate. Y: Broiler chickens fed with basal diet supplemented with 100 mg/kg yeast cell wall polysaccharides

^a,b^In the same row, values without common superscripts differ significantly (*P* < 0.05, *n* = 6)

### Lgr5 and Wnt/β-catenin signaling pathway related gene expression

As listed in Table 4**,** BMD notably decreased ileal mucosa β-catenin mRNA level relative to the control on d 21 (*P* < 0.05). On the contrary, on d 42, BMD remarkably up-regulated the expression of ISC markers Lgr5 and Wnt/β-catenin signaling pathway related genes axis inhibition protein 2 (*Axin2*) as well as Cyclin D1, compared with the control (*P* < 0.05). However, YCWP administration markedly upregulated ileal mucosal Wnt3 and β-catenin gene expression levels compared with the control and the BMD group at d 21 (*P* < 0.05). At 42 d of age, YCWP upregulated β-catenin mRNA level (*P* < 0.05), whereas the expression levels of *Axin2*, and Cyclin D1 were significantly downregulated by YCWP as compared to that of the BMD group (*P* < 0.05).

**Table 4:** Effects of yeast cell wall polysaccharides on Lgr5 and Wnt/β-catenin signaling pathway related genes expression in the ileum of broilers

Items	C	A	Y	SEM	*P*-value
d 21					
*Lgr5*	1.00	0.65	1.04	0.087	0.136
Wnt3	1.00^b^	0.68^b^	1.41^a^	0.095	0.002
β-Catenin	1.00^b^	0.56^c^	1.65^a^	0.148	0.003
*Axin2*	1.00	0.53	0.69	0.093	0.111
Cyclin D1	1.00	0.96	1.10	0.084	0.795
d 42					
*Lgr5*	1.00^b^	2.15^a^	1.73^ab^	0.191	0.034
Wnt3	1.00	0.84	1.22	0.075	0.119
β-Catenin	1.00^b^	0.89^b^	1.48^a^	0.088	0.005
*Axin2*	1.00^b^	3.36^a^	1.28^b^	0.283	< 0.001
Cyclin D1	1.00^b^	1.91^a^	0.99^b^	0.119	< 0.001

*Lgr5* Leucine-rich repeat containing G protein-coupled receptor, *Axin2* Axis inhibition protein 2. C: Broiler chickens fed with basal diet. A: Broiler chickens fed with the basal diet supplemented with 50 mg/kg bacitracin methylene disalicylate. Y: Broiler chickens fed with basal diet supplemented with 100 mg/kg yeast cell wall polysaccharides

^a–c^In the same row, values without common superscripts differ significantly (*P* < 0.05, *n* = 6)

### Intestinal cell biomarkers and intestinal tight junction protein gene expression

As illustrated in Table 5, on d 21, compared with the control, BMD significantly suppressed the gene expression of Paneth cells marker lysozyme, occludin and fatty acid binding protein 2 (*FABP-2*), while chicken fed with YCWP displayed higher marker of proliferation Ki-67 (*Mki67*) and lower lysozyme mRNA levels (*P* < 0.05). Nevertheless, relative to the BMD group, YCWP showed higher *Mki-67* and occludin gene expression (*P* < 0.05), whereas notably downregulated the lysozyme mRNA abundance (*P* < 0.05). On d 42, as compared with the control, BMD remarkably decreased the expression of goblet cell marker Mucin-2, lysozyme, occludin and *FABP-2* genes, but notably increased the mRNA abundance of E-cadherin genes in the ileum (*P* < 0.05). Nevertheless, compared with the BMD group, YCWP addition significantly upregulated Mucin-2, lysozyme, occludin and *FABP-2* gene expression, whereas remarkably downregulated E-cadherin gene expression in the ileum at d 42 (*P* < 0.05).

**Table 5:** Effect of yeast cell wall polysaccharides on intestinal cells proliferation and differentiation-related gene and barrier-related gene expression in the ileum of broilers

Items	C	A	Y	SEM	*P*-value
d 21					
Villin	1.00	0.62	0.97	0.078	0.093
*Mki67*	1.00^b^	0.83^b^	1.90^a^	0.148	0.001
Mucin-2	1.00	1.00	1.19	0.073	0.486
E-cadherin	1.00	1.09	1.10	0.129	0.940
Chromogranin A	1.00	0.73	1.32	0.108	0.069
Lysozyme	1.00^a^	0.70^b^	0.46^c^	0.060	< 0.001
Occludin	1.00^a^	0.75^b^	0.93^a^	0.037	0.011
Claudin-1	1.00	0.78	1.19	0.093	0.206
*FABP-2*	1.00^a^	0.31^b^	0.65^ab^	0.072	< 0.001
d 42					
Villin	1.00	1.29	1.00	0.074	0.184
*Mki67*	1.00	1.09	1.04	0.070	0.882
Mucin-2	1.00^a^	0.51^b^	1.22^a^	0.114	0.025
E-cadherin	1.00^b^	3.97^a^	1.38^b^	0.409	0.001
Chromogranin A	1.00	1.01	0.94	0.066	0.907
Lysozyme	1.00^a^	0.33^b^	0.89^a^	0.117	0.037
Occludin	1.00^a^	0.75^b^	1.13^a^	0.056	0.011
Claudin-1	1.00	1.20	1.03	0.061	0.352
*FABP-2*	1.00^a^	0.47^b^	0.89^a^	0.082	0.013

*Mki67* Marker of proliferation Ki67, *FABP-2* Fatty acid-binding protein 2. C: Broiler chickens fed with basal diet. A: Broiler chickens fed with the basal diet supplemented with 50 mg/kg bacitracin methylene disalicylate. Y: Broiler chickens fed with basal diet supplemented with 100 mg/kg yeast cell wall polysaccharides

^a–c^In the same row, values without common superscripts differ significantly (*P* < 0.05, *n* = 6)

### Goblet cell counts

**Fig. 2:**
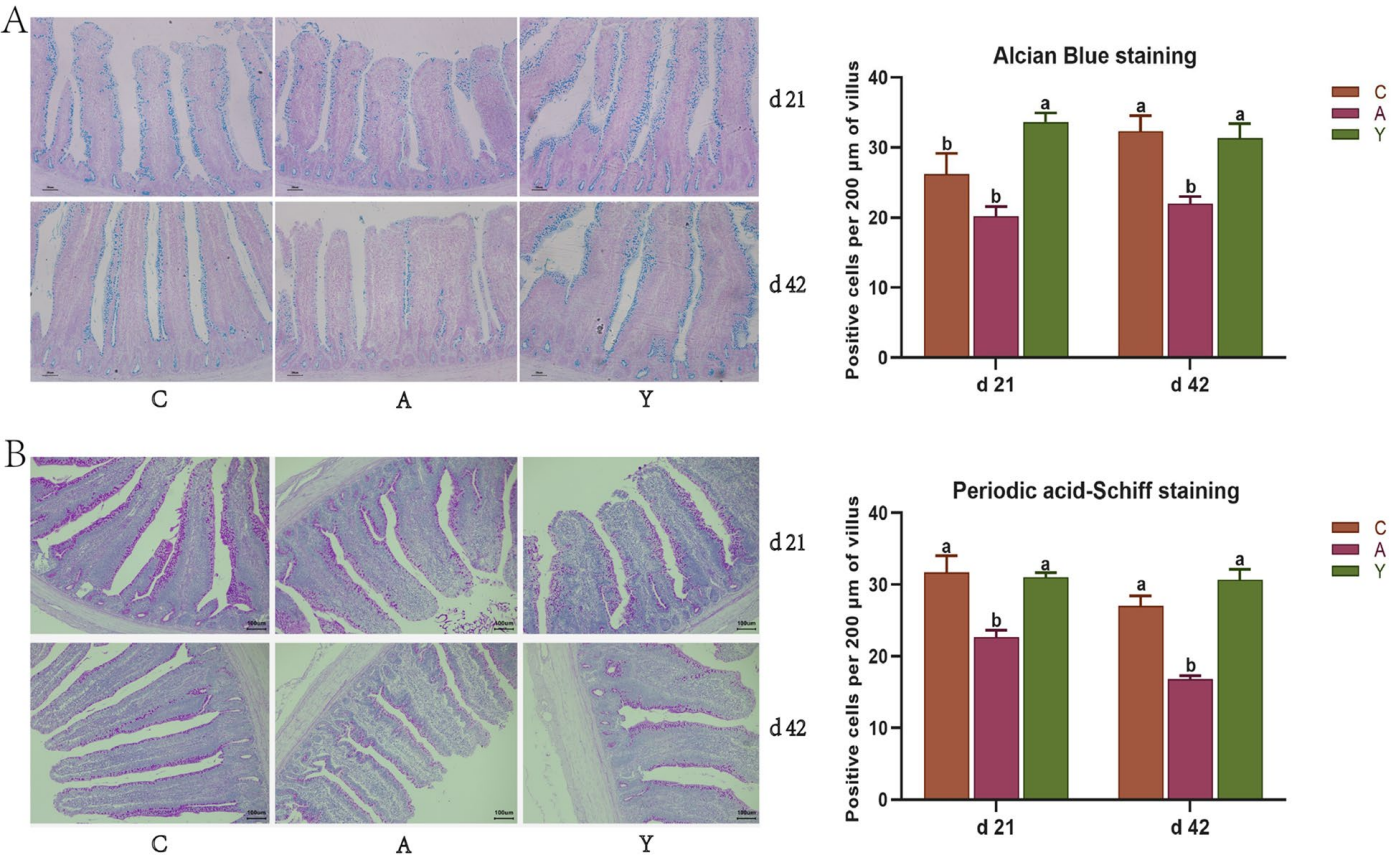
Effect of yeast cell wall polysaccharides on ileal mucin secreting cell amounts of chickens. **A** Alcian Blue stained the acidic mucus secreting goblet cell (× 400 magnification; scale bar: 100 μm). **B** Periodic Acid-Schiff stain the neutral mucus secreting goblet cell (× 400 magnification; scale bar: 100 μm). ^a,b^Different letters on the bar charts indicate significant differences (*P* < 0.05, *n* = 6). C: Broiler chickens fed with basal diet. A: Broiler chickens fed with the basal diet supplemented with 50 mg/kg bacitracin methylene disalicylate. Y: Broiler chickens fed with basal diet supplemented with 100 mg/kg yeast cell wall polysaccharides

Results of AB and PAS staining showed that BMD significantly decreased the number of goblet cells producing acidic and neutral mucins in the ileum at d 21 and d 42. Contrary to the results of BMD, YCWP administration remarkably increased ileal goblet cells numbers at d 21 and d 42 (Fig. 2, *P* < 0.05), which was similar to the control. Namely, YCMP administration notably promoted the intestinal stem cell to differentiate into the mature goblet cells, resulting in increasing intestinal development and defensive ability.

### Intestinal mucosal immune function

As illustrated in Table 6, on d 21, YCMP remarkably upregulated major histocompatibility complex class II (*MHC*-*II*), Toll-like receptors (*TLR*)-1 and 6 mRNA levels compared with the control (*P* < 0.05). Relative to the BMD group, YCWP significantly upregulated ileal mucosal *MHC-II*, *TLR-1*, *TLR-2* and *TLR-6* mRNA levels (*P* < 0.05).

**Table 6:** Effect of yeast cell wall polysaccharides on intestinal immune related marker genes expression in the ileum of broilers

Items	C	A	Y	SEM	*P*-value
d 21					
*CD80*	1.00	1.01	1.18	0.037	0.074
*CD83*	1.00	0.77	1.09	0.090	0.357
*CD86*	1.00	0.84	1.11	0.069	0.295
*MHC-I*	1.00	0.68	0.94	0.068	0.134
*MHC-II*	1.00^b^	0.75^b^	2.31^a^	0.220	0.002
*TLR-1*	1.00^b^	1.03^b^	1.94^a^	0.133	0.001
*TLR-2*	1.00^ab^	0.77^b^	1.28^a^	0.075	0.011
*TLR-6*	1.00^b^	0.92^b^	1.81^a^	0.126	0.001
*IL-1β*	1.00	1.03	0.83	0.105	0.739
*iNOS*	1.00	1.07	1.23	0.085	0.550
d 42					
*CD80*	1.00	1.16	0.99	0.055	0.366
*CD83*	1.00^a^	0.66^b^	0.86^a^	0.043	0.001
*CD86*	1.00	0.84	0.94	0.066	0.668
*MHC*-*I*	1.00^a^	0.36^b^	1.08^a^	0.088	< 0.001
*MHC*-*II*	1.00	1.23	1.15	0.110	0.707
*TLR-1*	1.00	1.45	1.06	0.092	0.085
*TLR-2*	1.00^b^	3.00^a^	0.92^b^	0.284	< 0.001
*TLR-6*	1.00^b^	3.79^a^	1.37^b^	0.368	< 0.001
*IL-1β*	1.00	1.21	0.56	0.368	0.165
*iNOS*	1.00^b^	2.24^a^	1.05^b^	0.168	< 0.001

*CD* Cluster of differentiation, *MHC* Major histocompatibility complex, *TLR* Toll-like receptors, *IL* interleukin, *iNOS* Inducible nitric oxide synthase. C: Broiler chickens fed with basal diet. A: Broiler chickens fed with the basal diet supplemented with 50 mg/kg bacitracin methylene disalicylate. Y: Broiler chickens fed with basal diet supplemented with 100 mg/kg yeast cell wall polysaccharides

^a,b^In the same row, values without common superscripts differ significantly (*P* < 0.05, *n* = 6)

**Fig. 3:**
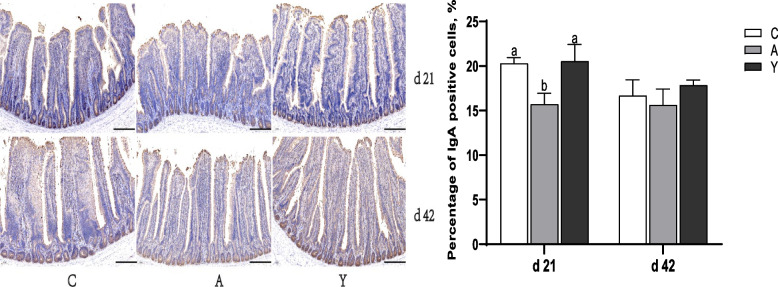
Effect of yeast cell wall polysaccharides on ileal IgA positive cells of chickens (× 400 magnification; scale bar: 100 μm). ^a,b^Different letters on the bar charts indicate significant differences (*P* < 0.05, *n* = 6). C: Broiler chickens fed with basal diet. A: Broiler chickens fed with the basal diet supplemented with 50 mg/kg bacitracin methylene disalicylate. Y: Broiler chickens fed with basal diet supplemented with 100 mg/kg yeast cell wall polysaccharides

At 42 d of age, compared with the control, BMD significantly increased *TLR-2*, *TLR-6* and inducible nitric oxide synthase (*iNOS*) mRNA abundance, but notably decreased cluster of differentiation (*CD*)83 and *MHC*-*I* mRNA levels (*P* < 0.05). Relative to the BMD group, YCWP significantly upregulated ileal mucosa *MHC*-*I* and *CD83* mRNA levels, markedly downregulated *TLR-2*, *TLR-6* and *iNOS* expression (*P* < 0.05). As shown in Fig. 3, BMD treatment significantly decreased the amount of ileal IgA-producing positive cells, compared with control group, while YCWP addition obviously increased the density of ileal IgA-producing positive cells at d 21 (*P* < 0.05), showing that BMD inhibited intestinal germinal centers to produce IgA positive B cells, while YCWP could stimulated the secretion of intestinal IgA.

### Cecal microbiome

**Fig. 4:**
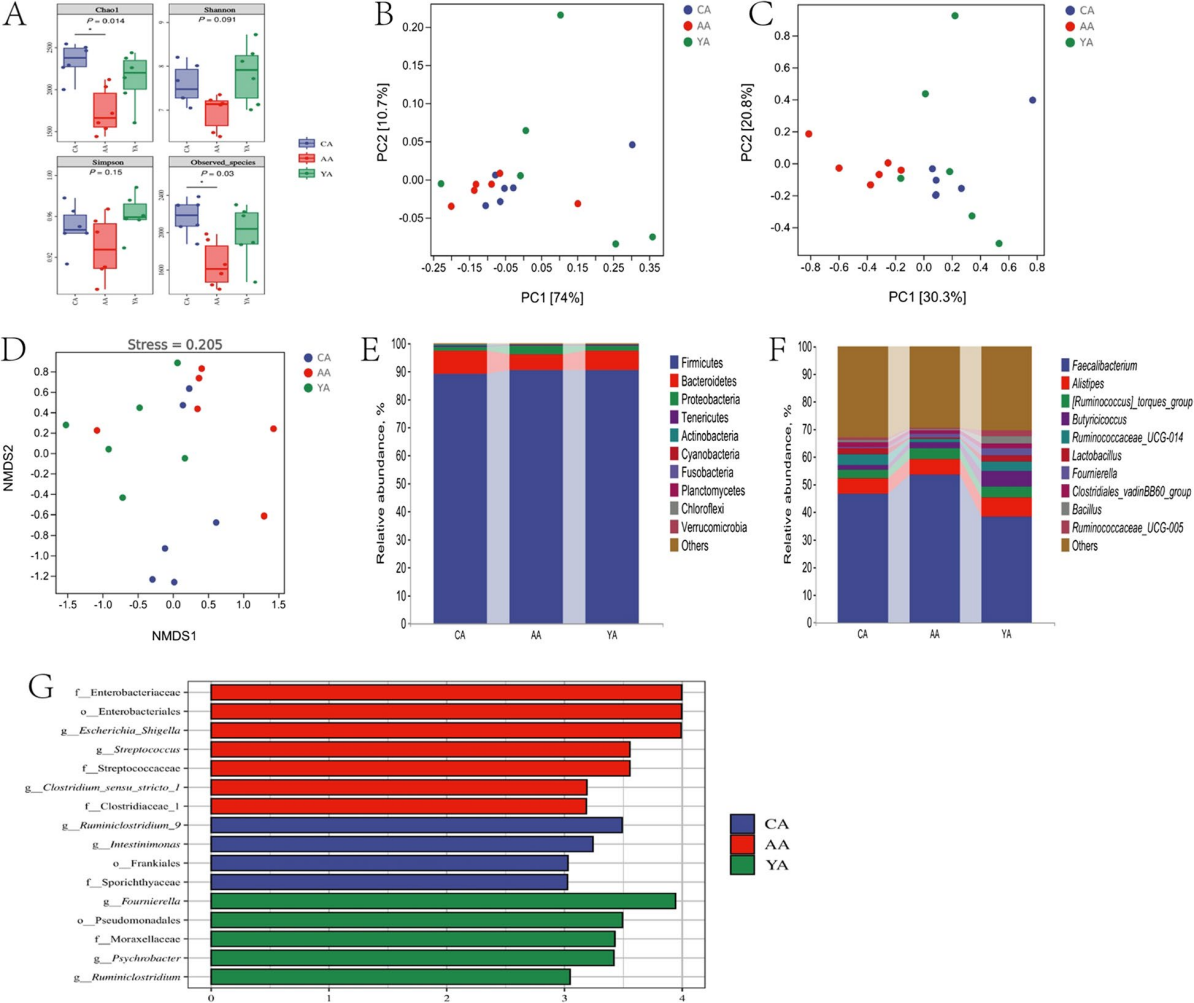
Differences in bacterial community diversity, richness, and structures in the cecum of chickens at d 21. **A** Community diversity and richness. **B** PCA analysis. **C** OPLS-DA analysis. **D** NMDS analysis. **E** Microbial composition at the phylum level. **F** Microbial composition at the genus level. **G** Histogram of LDA value distribution between groups. * means significant difference between groups (*P* < 0.05, *n* = 6). CA: Broiler chickens fed with basal diet. AA: Broiler chickens fed with the basal diet supplemented with 50 mg/kg bacitracin methylene disalicylate. YA: Broiler chickens fed with basal diet supplemented with 100 mg/kg yeast cell wall polysaccharides

**Fig. 5:**
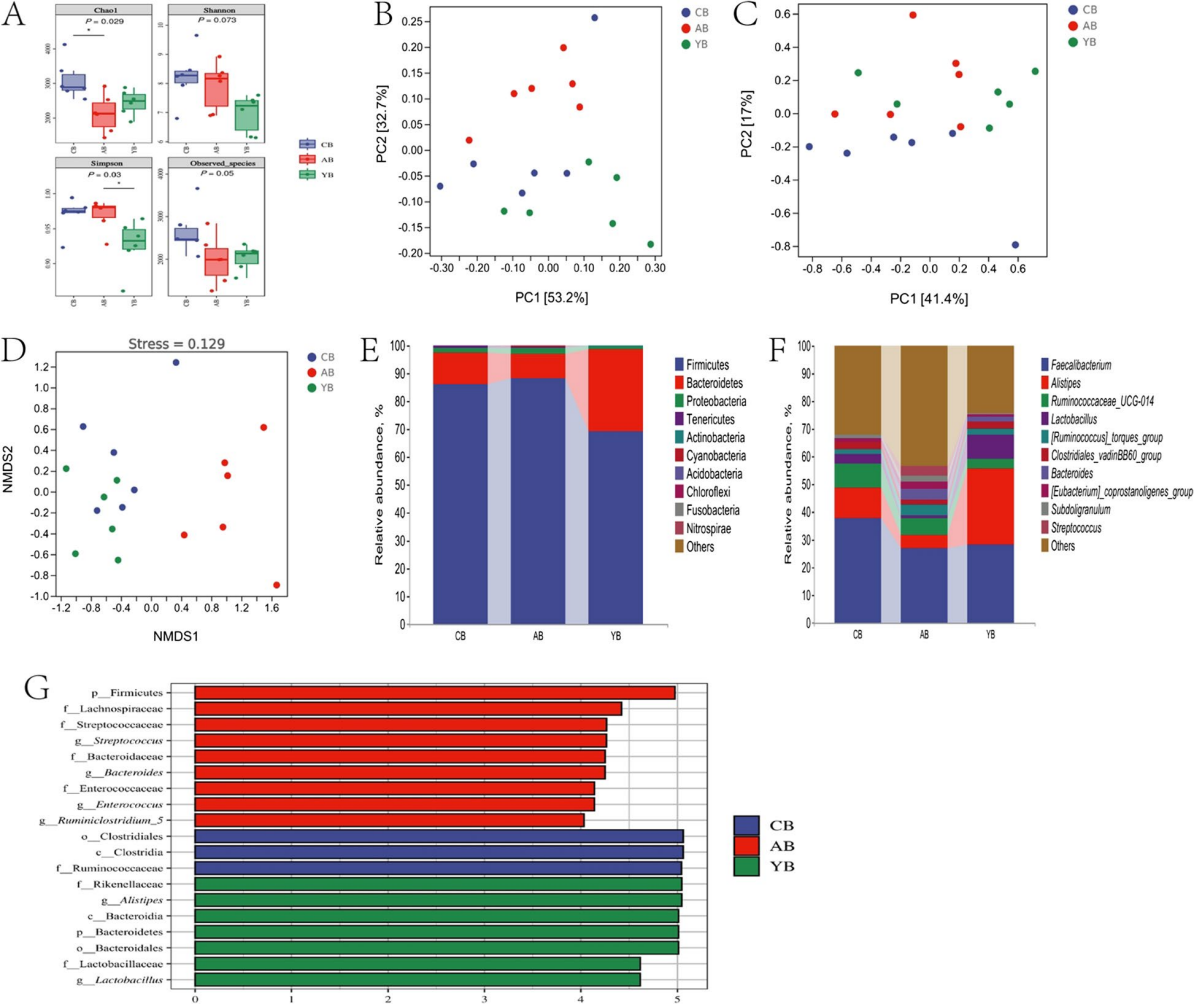
Differences in bacterial community diversity, richness, and structures in the cecum of chickens at d 42. **A** Community diversity and richness. **B** PCA analysis. **C** OPLS-DA analysis. **D** NMDS analysis. **E** Microbial composition at the phylum level. **F** Microbial composition at the genus level. **G** Histogram of LDA value distribution between groups. * means significant difference between groups (*P* < 0.05, *n* = 6). CB: Broiler chickens fed with basal diet. AB: Broiler chickens fed with the basal diet supplemented with 50 mg/kg bacitracin methylene disalicylate. YB: Broiler chickens fed with basal diet supplemented with 100 mg/kg yeast cell wall polysaccharides

As revealed in Fig. 4A, at 21 d of age, BMD decreased the Chao1 and observed species indexes (*P* < 0.05). Orthogonal partial least squares-discriminant analysis (OPLS-DA) analysis found that cecal microbial community of the control group and the YCWP group clustered together, but was obviously separated from that of the BMD group (Fig. 4C). As showed in Fig. 4E, F and Table S2, the cecal microbiota was dominated by Firmicutes, Bacteroidetes, Proteobacteria, Tenericutes and Actinobacteria at phylum level and *Faecalibacterium*, *Alistipes*, *[Ruminococcus]_torques_group*, *Butyricicoccus* as well as *Ruminococcaceae_UCG-014* at genus level of chicken at d 21. Statistical analysis showed that antibiotic BMD decreased *Ruminococcaceae_UCG-014* relative abundance at d 21 compared with the control (*P* < 0.05). LEfSe analysis found that antibiotic BMD treatment enriched relative abundances of the genus *Escherichia_Shigella*, *Clostridium_sensu_stricto_1* as well as *Streptococcus* in the cecum content of chickens at d 21 (Fig. 4G, *P* < 0.05). However, relative abundance of *Fournierella*, *Psychrobacter* and *Ruminiclostridium* were up-regulated after YCWP administration at d 21(*P* < 0.05). Likewise, at d 42, BMD significantly decreased the Chao1 index of cecal microbiota of chickens (Fig. 5A, *P* < 0.05). Strangely, YCWP also reduced the Simpson index (*P* < 0.05). Principal component analysis (PCA) and non-metric multidimensional scaling (NMDS) analysis noted that the microbial community of the BMD group was obviously separated from the other groups (Fig. 5B and D). As showed in Figs. 5E, 4F and Table S2, the cecal microbiota was dominated by Firmicutes, Bacteroidetes, Proteobacteria, Tenericutes and Actinobacteria at phylum level and *Faecalibacterium*, *Alistipes*, *Ruminococcaceae_UCG-014*, *Lactobacillus* as well as [*Ruminococcus*]*_torques_group* at genus level of chicken at d 42. Statistical analysis showed that BMD increased [*Ruminococcus*]*_torques_group* relative abundance at d 42 (*P* < 0.05). YCWP decreased the relative abundance of Firmicutes and Proteobacteria, while enriched Bacteroidetes relative abundance at phylum level (*P* < 0.05). LEfSe analysis displayed that *Streptococcus*, *Bacteroides*, *Enterococcus* and *Ruminiclostridium_5* were enriched in the BMD group (Fig. 5G, *P* < 0.05). *Alistipes* and *Lactobacillus* were enriched in chickens fed with YCWP (*P* < 0.05).

### Correlation analysis

**Fig. 6:**
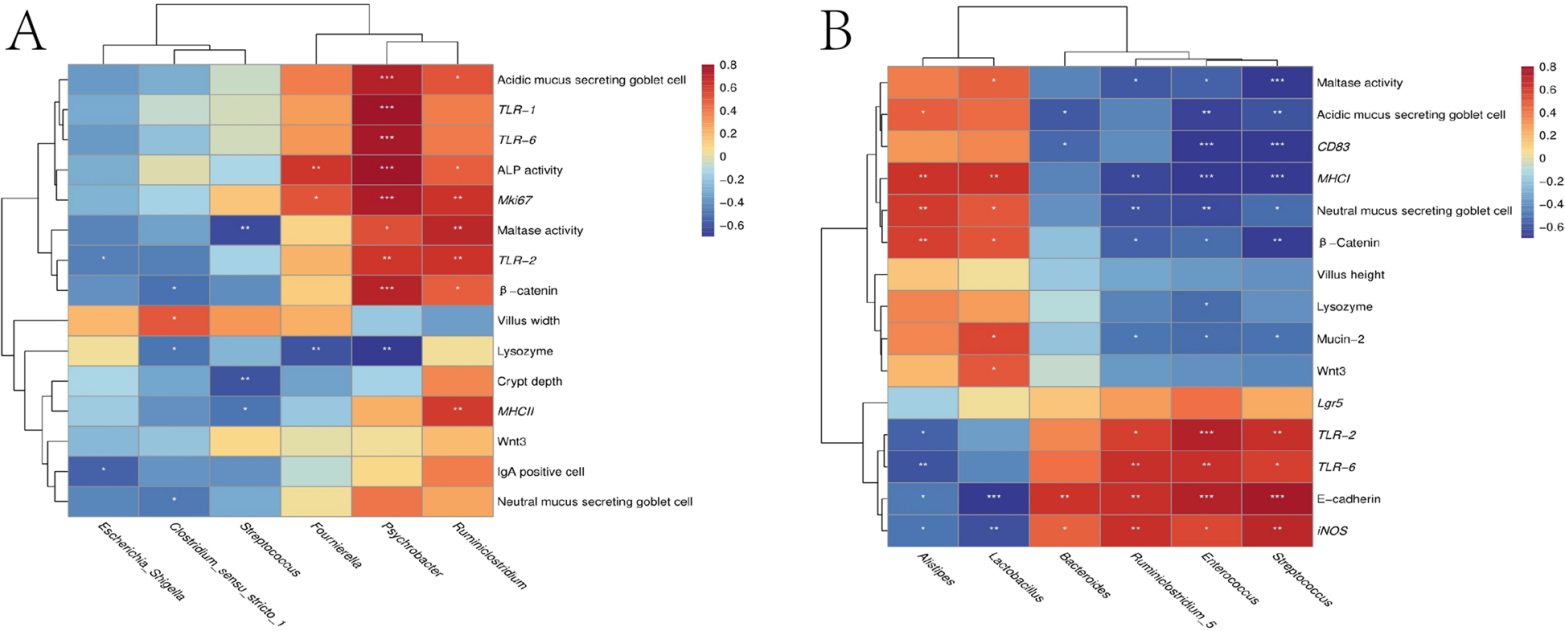
Correlation analysis between the relative abundance of different genera and gut development-related indexes of chickens at (**A**) d 21 and (**B**) d 42. Colors refer to the degree of correlation. ^*^0.01 < *P* < 0.05, ^**^0.001 < *P* < 0.01, ^***^P < 0.001

Spearman correlation analysis was conducted to clarify the relationship among gut microbiota and the intestinal development related index. On d 21, the abundance of *Escherichia_Shigella* was markedly negatively correlated with *TLR-2* mRNA level and IgA positive cell amount (*P* < 0.05). *Clostridium_sensu_stricto_1* was inversely related with the expression of β-catenin and lysozyme as well as neutral mucus secreting goblet cells counts while positively with villi width (*P* < 0.05). And *Streptococcus* was negatively correlated with crypt depth, maltase activity, and β-catenin, lysozyme, Mucin-2 and *MHC*-*II* mRNA abundance (Fig. 6A, *P* < 0.05). *Psychrobacter* relative abundance was positively related with most of gut development-related indexes such as ALP and maltase activity, β-catenin*,* *Mki67*, *TLR-1*, *TLR-2*, *TLR-6*, acidic mucus secreting goblet cells counts, but negatively with lysozyme mRNA levels (*P* < 0.05). *Fournierella* was negatively with lysozyme mRNA levels but positively related with ALP activity and *Mki67* levels (*P* < 0.05). *Ruminiclostridium* was positively correlated with ALP and maltase activity, β-catenin*, Mki67*, *MHC*-*II*, *TLR-2* and acidic mucus secreting goblet cells counts (*P* < 0.05).

On d 42, the relative population of *Streptococcus*, *Bacteroides*, *Enterococcus* and *Ruminiclostridium_5*, showed positive correlation with *iNOS* and E-cadherin expression (Fig. 6B), while showed negative correlation with maltase activity, goblet cell number, and *CD83*, *MHC*-*I* and β-catenin gene expression (*P* < 0.05). On the contrary, *Alistipes* and *Lactobacillus* had negative impacts on E-cadherin and *iNOS* expression but positive on neutral mucus secreting goblet cell number, β-catenin and *MHC*-*I* expression (*P* < 0.05). Moreover, *Lactobacillus* displayed a positive facilitating effect on the maltase activity, Mucin-2 and Wnt3 mRNA abundances (*P* < 0.05).

## Discussion

Crypt harbored ISCs could proliferate, polarize into diverse cell lineages including enterocytes, endocrine cells, Paneth cells and goblet cells and migrate up the villus [10]. The increase in crypt depth may represent an increase in the number of proliferating stem cells [15]. In addition, villus height, the activity of intestinal ALP and brush border disaccharidase (maltase, surcease) are also key biomarkers of enterocyte differentiation and maturation [37]. This study first investigated the impact of dietary YCWP on intestinal morphology development and intestinal cells maturity-related enzyme activities. Our data showed that BMD significantly shortened crypt depth at d 21 and villus height at d 42, decreased maltase activity at both d 21 and d 42 compared with the control group, which was consistent with previous observation [38]. However, YCWP supplementation promoted intestinal development of chicken, as reflected by increased ileal villus height at d 21 and d 42 along with crypt height at d 21, enhanced ALP at d 21 and maltase activities at both d 21 and d 42 relative to BMD group. The results were in consistent with previous findings that yeast-derived carbohydrates such as mannooligosaccharides, β-glucan and their combination supplementation, or yeast cell wall products increased intestinal villus height, and/or increased the ratio of villus height to crypt depth in broiler chickens [24, 39–42]. So, our data suggested that chickens continuously supplemented with antibiotics BMD had adverse influences while YCWP had beneficial effects on intestinal morphological development.

Wnt/β-catenin signaling pathways play crucial role in modulating villus formation, epithelial renewal and intestinal development by affecting the proliferation and differentiation activity of intestinal stem cells [43]. Our results indicated that BMD treatment significantly downregulated the expression of β-catenin on d 21 and d 42. Inversely, YCWP administration markedly upregulated ileal mucosal gene expression levels of Wnt3, *Mki67* on d 21 and β-catenin at d 21 and d 42. In consistent with our findings, previous studies had demonstrated that antibiotic treatment blocked the Wnt/β-catenin signaling pathway, decreased lysozyme expression, goblet cell number and mucous layer thickness of chickens [44, 45]. Similarly, several studies had also indicated that YCWP could stimulate the Wnt/β-catenin pathway, elevate the crypt cell mitotic indices and induce advanced goblet cell density and mucus layer thickness of chicken [46, 47]. Thus, our data suggested that BMD administration blocked the activation of Wnt/β-catenin signaling pathway of chickens, while YCWP addition could promote intestinal development via stimulating Wnt/β-catenin signaling pathway of chickens. Surprisingly, a notable up-regulation in *Lgr5*, *Axin2* as well as Cyclin D1 mRNA levels was observed in the ileum of the BMD group at d 42. Lgr5 is a receptor expressed on crypt intestinal stem cells that responses to Wnt signals that construct and maintain the crypt stem cell niche [48]. Previous studies reported that continuous BMD addition disturbed the balance of intestinal microbiota, resulting in intestinal inflammation and compensatively enhancing the regenerative repair activity of Lgr5^+^ ISCs [49, 50]. Thus, we suggested that continuous BMD feeding did not activate Wnt/β-catenin pathway, only compensatively increased ISCs proliferation activity in later life of chickens, possibly due to intestinal inflammation.

Turnover and maturity of intestinal epithelium are strongly related with gut barrier defensive functions of broilers [51–53]. In this study, contrary to BMD group, YCWP addition not only significantly upregulated intestinal barrier related gene expression as well as intestinal epithelial cell proliferation marker *Mki67* mRNA abundance, but also increased the number of goblet cells in the ileum. Similar to our findings, previous studies had confirmed that yeast cell wall derived polysaccharide supplementation could positively alter the intestinal microenvironment via increasing acidic goblet cell density, Mucin-2 transcription, along with enhancing intestinal barrier function related gene expression in chickens [24, 42, 54]. Combined with above observation, it can be suggested that YCWP addition could boost intestinal epithelial barrier and defensive function, possibly by stimulating intestinal stem cells to proliferate and differentiate into mature functional cells in a Wnt/β-catenin dependent activation manner.

The development and maturation of immune system of chickens was important for the gut defensive function of chickens. MHC-I and MHC-II, co-stimulatory molecules CD80 and CD86, are key molecules involved in antigen presentation upon macrophage and dendritic cells activation along with host innate immunity [55]. Secretory immunoglobulin A (sIgA) was secreted by plasma cells which was associated with intestinal mucosal immunity and gut health [56]. In this study, our data indicated that continuous BMD treatment compromised intestinal innate immunity of chickens via downregulating antigen presenting cell markers *MHC-I*, *MHC-II* and *CD83* mRNA levels and decreasing IgA-positive plasma cell numbers in the ileum. These data suggested that BMD administration might delay the development of intestinal innate mucosal system of chickens. TLR signaling pathway play key role in modulating innate immune responses [57, 58]. Interestingly, upregulated *TLR-2* and *TLR-6 *and *iNOS* mRNA abundance was found in the BMD group in the later stage, indicating that continuous BMD might induce intestinal inflammation possibly by activating TLR-mediated inflammatory response. A previous study reported that continuous antibiotics BMD treatment enriched the abundance of potentially harmful gut bacteria, resulting in the overproduction of intestinal pro-inflammatory molecules of broilers [49]. Thus, we suggested that intestinal inflammatory response and intestinal barrier disruption observed in continuous BMD-treated chickens in our study was possibly due to the proliferation of intestinal potential harmful bacteria. Gut inflammation could obviously stimulate the over-proliferation of intestinal stem cells and activated Wnt/β-catenin signaling pathway. So, this finding obtained in BMD-treated chickens might provide a reasonable explanation for the activation of Wnt/β-catenin signaling pathway after continuous BMD administration. However, continuous addition of YCWP remarkably upregulated innate immune cell markers *MHC*-*II*, *TLR-1* and *TLR-6*, and the amount of ileal IgA-producing plasma cells at d 21 together with innate immune cell markers *MHC*-*I* and *CD83* mRNA levels at d 42 compared with the control and the BMD group. Consistent with our findings, previous researches had showed that YCWP could strengthen intestinal innate immunity including cellular and humoral immune functions in broiler chickens [20, 59]. Thus, our data indicated that YCWP supplementation could enhance intestinal immune function by increasing antigen presenting cell activities, activating TLR-mediated signaling pathway along with boosting sIgA levels, which was opposite to antibiotic BMD.

Emerging evidence had been proved that gut microbiota is critical for the development of intestinal structure and morphology, mucosal and systemic immunity, intestinal barrier function, nutrient digestion and absorption, along with resistance against colonization or overgrowth by enteric pathogens [60]. Thus, targeting the modulation of microbiota using effective non-antibiotics modulator develops as a promising strategy to promote the gut development and immune system maturity. In the study, the impacts of YCWP and BMD on colonization of cecal microbiota, and spearman correlation analysis among gut microbiome and intestinal development parameters were established. Microbiome analysis of the cecum contents showed that continuous antibiotics BMD inclusion enriched potential harmful bacteria such as *Escherichia_Shigella*, and *Clostridium_sensu_stricto_1* as well as *Streptococcus* relative abundances at d 21, expanded *Streptococcus*, *Bacteroides*, *Enterococcus* and *Ruminiclostridium_5* relative population at d 42 with the extension period of BMD, while decreasing Chao1 index of cecal microbiota and potential beneficial bacteria *Ruminococcaceae_UCG-014* relative abundance on d 21 in chickens, displayed a retardative microbiota succession and unhealthy gut microbiota composition which may due to strong antibacterial properties of BMD [49, 61]. Correlation analysis results showed differential microbiota including *Escherichia_Shigella*, *Clostridium_sensu_stricto_1*, *Streptococcus* and *Enterococcus* enriched in antibiotic BMD group had negative correlation with intestinal development and maturation related markers, which was consistent with previous studies [62, 63]. *Escherichia_Shigella*, *Streptococcus* and *Enterococcus* are usually known to be opportunistic pathogens inducing intestinal inflammation response in chickens [64], which provided reasonable explanation for our speculation above that intestinal inflammation induced by continuous BMD treatment. However, dietary YCWP continuous supplementation notably upregulated relative abundance of *Fournierella*, *Psychrobacter* and *Ruminiclostridium* on d 21, and enriched *Bacteroidetes*, *Alistipes* and *Lactobacillus* relative abundance on d 42. Correlation analysis noted that microbiota enriched in YCWP group such as *Psychrobacter*, *Fournierella*, *Ruminiclostridium*, *Alistipes* and *Lactobacillus* displayed positive relation with intestinal development and maturation as well as innate immune related markers. Moreover, *Lactobacillus* relative abundance was positively related with Wnt3 mRNA abundance. *Fournierella* was reported as a crucial driver for chicken immune development [65]. *Ruminiclostridium* had the ability degrading gut cellobiose, producing short chain fatty acids and promoting chicken ISCs polarization and maintaining gut immune homeostasis [66]. Additionally, *Alistipes* was positively associated with goblet cell numbers and enhanced intestinal Mucin-2 secretion [67]. *Lactobacillus* was shown to influence intestinal development and maturity, such as promoting ISC differentiation into goblet cells of broilers through activating Wnt signaling pathway [68–70], besides positively involved in immuno-regulation and intestinal health [71, 72]. The diversity of microbiota increases with age, and higher microbial diversity and higher relative population of intestinal potential beneficial bacteria are considered to be mature intestinal microbiota which possess advanced resistance against infection [73]. Based on these findings, we suggested that continuous antibiotic BMD exposure delayed or disturbed the development of a healthy gut microbiome through promoting the growth of intestinal potential pathogens in chickens. While YCWP continuous inclusion accelerated intestinal development and maturation of a healthy gut microbiome, possibly by increasing intestinal potential beneficial microbiota abundance, which provide a reasonable explanation for the increases in intestinal ISCs proliferation and differentiation, as well as the enhancement of intestinal innate immunity and gut barrier function observed in YCWP-treated chickens. Taken together, we suggested YCWP facilitated yet antibiotic BMD delayed intestinal development and maturation in chickens, possibly due to different regulated pattern in gut microbiome. Further studies were required to confirm the promoting effects of YCWP on gut development and maturation through fecal microbiota transplant. Collectively, the study provides a valuable vision to promote chicken intestinal development and increase disease resistance by using effective non-antibiotics substances.

## Conclusion

In summary, dietary YCWP inclusion could accelerate intestinal development and maturity, possibly through promoting intestinal stem cells to differentiate into mature intestinal epithelial cells, enhancing intestinal innate immunity and barrier defensive function in broilers. The improving effects induced by YCWP possibly is involved in improved intestinal microbiome and the activation of Wnt/β-catenin signaling pathway of intestinal stem cells.

## Supplementary Information

Additional file 1. Table S1. Sequences of the oligonucleotide primers used for quantitative real-time PCR. Table S2. Effect of bacitracin methylene disalicylate and yeast cell wall polysaccharides on microbial composition in the cecum of broilers.

## Data Availability

The 16S rRNA gene sequencing data generated and analyzed during the current study are available in the NCBI primary data archive with accession number PRJNA 987,872. This data can be found here: https://www.ncbi.nlm.nih.gov/bioproject/987872.

## Abbreviations

AB: Alcian Blue
ALP: Alkaline phosphatase
BMD: Bacitracin methylene disalicylate
CD: Cluster of differentiation
FABP-2: Fatty acid binding protein 2
IgA: Immunoglobulin A
iNOS: Inducible nitric oxide synthase
ISC: Intestinal stem cell
LDA: Linear discriminant analysis
LEfSe: Linear discriminant analysis combined effect size
Lgr5: Leucine-rich repeat-containing G protein-coupled receptor 5
MHC: Major histocompatibility complex
Mki67: Marker of proliferation Ki-67
NMDS: Non-metric multidimensional scaling
OPLS-DA: Orthogonal partial least squares-discriminant analysis
OTUs: Operational taxonomic units
PAS: Periodic acid-Schiff
PCA: Principal component analysis
RT-qPCR: Quantitative real-time PCR
TLR: Toll-like receptors
VSA: Villus surface area
YCWP: Yeast cell wall polysaccharides
