# Supplementary material for: Yeast cell wall polysaccharides accelerate yet in-feed antibiotic delays intestinal development and maturation via modulating gut microbiome in chickens
Source: J Anim Sci Biotechnol. 2025 Jan 25;16:14. doi: 10.1186/s40104-024-01145-x (PMC11763161; doi:10.1186/s40104-024-01145-x)
Supplement: Supplementary file 1 — Additional file 1. Table S1. Sequences of the oligonucleotide primers used for quantitative real-time PCR. Table S2. Effect of bacitracin methylene disalicylate and yeast cell wall polysaccharides on microbial composition in the cecum of broilers. [file 40104_2024_1145_MOESM1_ESM.docx]

**Table S1** Sequences of the oligonucleotide primers used for quantitative real-time PCR

| **Gene name** | **Primer sequence (5' to 3')** | **GenBank accession** |
| --- | --- | --- |
| β-actin | CAACACAGTGCTGTCTGGTGGTAC | L08165 |
|  | CTCCTGCTTGCTGATCCACATCTG |  |
| CD80 | CAGCAAGCCGAACATAGAAAGA | NM_001079739.2 |
|  | AGCAAACTGGTGGACCTGAGA |  |
| CD83 | GCTGACTTGCCTCGGGATT | XM_418929 |
|  | TCACTCCGCTATCCGTCTCA |  |
| CD86 | CTACCAGCCAGAGTGTCAGT | NM_001037839.1 |
|  | ATGGTTCCCAGTCAGCAGTC |  |
| MHCI | TCGAGCACAGGGAGCAA | NM_001044683.3 |
|  | TCAGCATAGGAAGCAGAACGA |  |
| MHCII | AGGTGCTGGTGGTGCTGGAG | NM_001044679.2 |
|  | GCCGTCTGAGCGACTTCTTGG |  |
| TLR-1 | AACCCGTTCAAGTGTTCGTG | NM_001007488.5 |
|  | GTTCCGCTCAAGTCTTCTGG |  |
| TLR-2 | ACCTTCTGCACTCTGCCATT | NM_001161650.3 |
|  | TGTGAATGAAGCACCGGTAA |  |
| TLR-6 | CCAGAAGACTTGAGCGGAACACAG | NM_001081709.4 |
|  | TCTCCTCTTCGTCTGCGTCCAC |  |
| IL-1β | ACTGGGCATCAAGGGCTA | NM_204524.2 |
|  | GGTAGAAGATGAAGCGGGTC |  |
| E-cadherin | ACTGGTGACATTATTACCGTAGCA | NM_001001615.2 |
|  | TAGCCACTATGACATCCACTCTGT |  |
| Chromogranin A | TGAATAAAGGGGACACTAAGG | XM_421330.8 |
|  | AGCTCAGCCAGGGATG |  |
| Lysozyme | CCCAGGCTCCAGGAACCT | NM_205281 |
|  | CACGCTCGCTGTTATGTCTGA |  |
| Villin | AGCACTTCCCCTAGGTACG | NM_001396564.1 |
|  | GCACCATCTCCATGTTCTCG |  |
| Mucin-2 | TTCATGATGCCTGCTCTTGTG | XM_040701667.1 |
|  | CCTGAGCCTTGGTACATTCTTGT |  |
| iNOS | GAACAGCCAGCTCATCCGATA | NM_204961.1 |
|  | CCCAAGCTCAATGCACAACTT |  |
| Lgr5 | TCAATACCTGAGCGTGCGTT | XM_425441.7 |
|  | TGTGAGTGTCAAACTCTCCAGAC |  |
| Wnt3 | GAAGCTGCGAGGTCAAGACT | NM_001171601.1 |
|  | TTGCACGTTCTGTCCCTTGT |  |
| β-catenin | ATTTGTGCGCTCCGTCAC | NM_205081.3 |
|  | ACCTTGTTCACGCAGTGG |  |
| Mki67 | ATTCGCATCCACTTGCCTCA | XM_015289038.4 |
|  | TGCTGAACATGAAGAACCTGC |  |
| Cyclin D1 | CCCGACGAGTTACTGCAAATG | NM_001396513.1 |
|  | GCGCACAGAGCCACAAAAG |  |
| Axin2 | GCAGCAAATCAAGGAGGATGAG | NM_204491.1 |
|  | GGGTTCACCATCTCCCGACT |  |
| Occludin | ACGGCAGCACCTACCTCAA | NM_205128.1 |
|  | GGGCGAAGAAGCAGATGAG |  |
| Claudin-1 | CATACTCCTGGGTCTGGTTGGT | NM_001013611.2 |
|  | GACAGCCATCCGCATCTTCT |  |
| FABP-2 | TGGAAGCAATGGGCGTGAAT | NM_001007923.1 |
|  | TGTCGATGGTACGGAAGTTGC |  |

CD, cluster of differentiation. MHC, major histocompatibility complex. TLR, Toll-like receptors. IL, interleukin. iNOS, inducible nitric oxide synthase. Lgr5, Leucine-rich repeat containing G protein-coupled receptor. Mki67, marker of proliferation Ki67. Axin2, axis inhibition protein 2. ZO-1, Zona occludens-1. Primers were synthesized by Sangon Biotech (shanghai) Co., Ltd.

**Table S2** Effect of bacitracin methylene disalicylate and yeast cell wall polysaccharides on microbial composition in the cecum of broilers

| **Items** | **C** | **A** | **Y** | **SEM^1^** | ***P* value** |
| --- | --- | --- | --- | --- | --- |
| d 21 |  |  |  |  |  |
| Phylum |  |  |  |  |  |
| Firmicutes | 89.28 | 90.22 | 90.59 | 1.574 | 0.470 |
| Bacteroidetes | 8.27 | 6.00 | 6.97 | 1.533 | 0.343 |
| Proteobacteria | 1.21 | 2.89 | 1.72 | 0.402 | 0.229 |
| Tenericutes | 0.45 | 0.34 | 0.36 | 0.088 | 0.641 |
| Actinobacteria | 0.35 | 0.21 | 0.07 | 0.046 | 0.119 |
| Genus |  |  |  |  |  |
| *Faecalibacterium* | 46.56 | 53.29 | 38.09 | 3.799 | 0.185 |
| *Alistipes* | 5.64 | 5.74 | 6.85 | 1.308 | 0.519 |
| *[Ruminococcus]_torques_group* | 3.03 | 4.06 | 4.08 | 0.559 | 0.484 |
| *Butyricicoccus* | 1.55 | 1.98 | 5.51 | 1.301 | 0.932 |
| *Ruminococcaceae_UCG-014* | 3.88^a^ | 1.25^b^ | 3.74^ab^ | 0.605 | 0.045 |
| d 42 |  |  |  |  |  |
| Phylum |  |  |  |  |  |
| Firmicutes | 86.23^a^ | 88.29^a^ | 69.06^b^ | 2.784 | 0.008 |
| Bacteroidetes | 11.35^b^ | 8.74^b^ | 29.59^a^ | 2.880 | 0.006 |
| Proteobacteria | 1.66^a^ | 2.19^a^ | 0.73^b^ | 0.220 | 0.005 |
| Tenericutes | 0.54 | 0.33 | 0.36 | 0.060 | 0.423 |
| Actinobacteria | 0.06 | 0.18 | 0.08 | 0.023 | 0.149 |
| Genus |  |  |  |  |  |
| *Faecalibacterium* | 37.61 | 26.97 | 28.01 | 3.377 | 0.372 |
| *Alistipes* | 10.84^ab^ | 4.80^b^ | 27.57^a^ | 2.987 | 0.003 |
| *Ruminococcaceae_UCG-014* | 8.71 | 5.79 | 3.30 | 1.204 | 0.065 |
| *Lactobacillus* | 3.55^ab^ | 0.88^b^ | 8.85^a^ | 1.253 | 0.006 |
| *[Ruminococcus]_torques_group* | 2.00^b^ | 4.26^a^ | 2.21^b^ | 0.404 | 0.014 |

^1^SEM, standard error of the mean. C broiler chickens fed with basal diet. A broiler chickens fed with the basal diet supplemented with 50 mg/kg bacitracin methylene disalicylate. Y broiler chickens fed with basal diet supplemented with 100 mg/kg yeast cell wall polysaccharides. ^a, b^ Means in the same column without common superscripts differ significantly (*P* < 0.05).
